# Natural *Schistosoma mansoni* Infection in the Wild Reservoir *Nectomys squamipes* Leads to Excessive Lipid Droplet Accumulation in Hepatocytes in the Absence of Liver Functional Impairment

**DOI:** 10.1371/journal.pone.0166979

**Published:** 2016-11-23

**Authors:** Kátia B. Amaral, Thiago P. Silva, Kássia K. Malta, Lívia A. S. Carmo, Felipe F. Dias, Mariana R. Almeida, Gustavo F. S. Andrade, Jefferson S. Martins, Roberto R. Pinho, Sócrates F. Costa-Neto, Rosana Gentile, Rossana C. N. Melo

**Affiliations:** 1 Laboratory of Cellular Biology, Department of Biology, Federal University of Juiz de Fora (UFJF), Juiz de Fora, MG, Brazil, 36036–900; 2 Laboratory of Plasmonic Nanostructures, Molecular Spectroscopy and Structure Group, Department of Chemistry, Federal University of Juiz de Fora (UFJF), Juiz de Fora, MG, Brazil, 36036–900; 3 Department of Physics, Federal University of Juiz de Fora (UFJF), Juiz de Fora, MG, Brazil, 36036–900; 4 Laboratory of Biology and Parasitology of Wild Reservoir Mammals, Oswaldo Cruz Foundation, Rio de Janeiro, Brazil; Universidade Guarulhos, BRAZIL

## Abstract

Schistosomiasis is a neglected tropical disease of a significant public health impact. The water rat *Nectomys squamipes* is one of the most important non-human hosts in the schistosomiasis mansoni transmission in Brazil, being considered a wild reservoir. Cellular mechanisms that contribute to the physiological adaptation of this rodent to the Schistosoma *mansoni* parasite are poorly understood. Here we identified, for the first time, that a hepatic steatosis, a condition characterized by excessive lipid accumulation with formation of lipid droplets (LDs) within hepatocytes, occurs in response to the natural *S*. *mansoni* infection of *N*. *squamipes*, captured in an endemic region. Significant increases of LD area in the hepatic tissue and LD numbers/hepatocyte, detected by quantitative histopathological and ultrastructural analyses, were paralleled by increased serum profile (total cholesterol and triglycerides) in infected compared to uninfected animals. Raman spectroscopy showed high content of polyunsaturated fatty acids (PUFAs) in the liver of both groups. MALDI-TOFF mass spectroscopy revealed an amplified pool of omega-6 PUFA arachidonic acid in the liver of infected animals. Assessment of liver functional activity by the levels of hepatic transaminases (ALT and AST) did not detect any alteration during the natural infection. In summary, this work demonstrates that the natural infection of the wild reservoir *N*. *squamipes* with *S*. *mansoni* elicits hepatic steatosis in the absence of liver functional harm and that accumulation of lipids, markedly PUFAs, coexists with low occurrence of inflammatory granulomatous processes, suggesting that lipid stores may be acting as a protective mechanism for dealing with the infection.

## Introduction

Schistosomiasis is an important neglected tropical disease caused by parasitic worms of the genus *Schistosoma*, with a significant socioeconomic impact [1]. Schistosomiasis is acquired when free-swimming parasitic larvae (cercariae), released by freshwater snails, penetrate the skin of people exposed to infested freshwater. Exposure to infection results from a lack of safe, alternative water sources for agricultural, domestic, and/or recreational activities (reviewed in [1, 2]).

The only species of *Schistosoma* that occurs in the Americas is *S*. *mansoni* [1]. Human infection with this parasite causes marked chronic morbidity with development of a granulomatous reaction and severe tissue inflammation, in particular within the liver and intestines, which can lead to life-threatening hepatosplenomegaly (reviewed in [3]). The transmission of *Schistosomiasis mansoni* occurs largely in Brazil, affecting million people with its advanced clinical forms. As part of the World Health Organization’s strategic plan for the period of 2010–2020, Brazil is one of the endemic countries that require intensification of preventive chemotherapy and implementation of complementary public-health measures with the aim of interrupting schistosomiasis transmission [1].

A key feature in the transmission of schistosomiasis mansoni is that some species of *Schistosoma* that infect humans also infect wild vertebrate hosts. These animals hold the infection, and in some cases, increase the probability that humans find these parasites in natural ecosystems, acting as wild reservoirs [4, 5]. The water rat *Nectomys squamipes* is considered one of the most important non-human hosts in the schistosomiasis mansoni transmission in Brazil, generally considered as a reservoir due to its semi-aquatic habits, wide geographic distribution, high susceptibility and abundance and tolerance to human presence [6–8]. This rodent from the Cricetidae family is found mainly in the Atlantic forest, from the Northeast to the South of Brazil. The species lives in close contact with fresh water collections, where the animals eliminate viable eggs, actively participating in the transmission dynamics of the parasite in natural environments [9].

Despite being highly susceptible to the *S*. *mansoni* infection, long-term field studies of *N*. *squamipes* populations, based on mark-recapture technique, showed no influence of this parasite on the survival and reproductive capacity of the water-rat [8, 10, 11]. Indeed, early studies have indicated that *N*. *squamipes* presents high compatibility in the host-parasite relationship, and that *N*. *squamipes* population parameters were not affected by the infection [8]. However, cellular mechanisms that contribute to the physiological adaptation of this rodent to the S. *mansoni* parasite are poorly understood.

By studying the liver histopathology of *N*. *squamipes* naturally infected with *S*. *mansoni* and captured in an endemic area in Brazil, we noticed that this target organ of the disease exhibited an apparent “hepatic steatosis” (fatty liver). Here, we identified for the first time, that the natural *S*. *mansoni* infection of *N*. *squamipes* elicits excessive lipid droplet (LD) formation within hepatocytes and increases serum lipid profile in parallel to a low incidence of inflammatory granulomatous response. Interestingly, these lipid alterations occur in the absence of liver functional impairment, suggesting that lipid accumulation may be acting as a protective mechanism for dealing with the infection.

## Materials and Methods

### Study area

Adult specimens of *N*. *squamipes* were captured in the rural areas of the Municipality of Sumidouro (22° 02' 46" South and 42° 41' 21" West), located in the mountainous region of the state of Rio de Janeiro, Brazil, an endemic area of human schistosomiasis and where the presence of this rodent has often been registered [9]. Capture transects were established in *Encanto* and *Pamparrão* localities along streams and irrigation channels, which constitute the habitat of this rodent. Tomahawk® traps measuring 40 cm x 64 cm x 12.7 cm were placed on the ground and baited with a mixture of peanut butter, banana, oat and bacon. [12]

### Identification of adult worms and parasite burden

Infected *N*. *squamipes* were identified by the presence of adult worms in mesenteric veins using perfusion of the portal-hepatic system [12]. For this procedure, a Brewer® perfusor, also known as Automatic Pipetting Machine (cat number 60480, model 40A, Scientific Equipment Products, MD, patent number 2148899) was used. Saline (1.8%) was inoculated through the right ventricle and the liquid obtained from the perfusion was filtered through a fine mesh fabric to retain the adult worms. Worms recovered from each infected animal were counted with the aid of a stereomicroscope. Male and female worms were morphologically identified and counted as previous work [8]. In addition to the presence of adult worms in the mesenteric veins, positivity was confirmed by parasite eggs found in stool tests [13].

### Experimental infection in mice

Swiss Webster mice aged 70 days were inoculated or not (18 mice per group) with a single inoculum of cercariae of *S*. *mansoni* (100 cercariae/mouse), LE strain. Cercariae were harvested from infected *Biomphalaria glabrata* snails, washed, counted, and injected subcutaneously into each mouse by an experienced technician. *S*. *mansoni* strain LE used in the experiments was originally isolated from a patient in Belo Horizonte, Brazil, and has been maintained in successive passages through *Biomphalaria glabrata* snails and hamsters (*Mesocricetus auratus*) at the Laboratory of Schistosomiasis (Department of Parasitology, UFMG, Brazil). Infected animals and respective uninfected controls from the same age were euthanized at 55 days or 120 days of infection. Infection was confirmed by findings of parasite eggs in the rodent feces at week five of infection [13].

### Collection of samples

Both naturally and experimentally infected animals and their respective uninfected controls were anesthetized, euthanized, and blood samples and organ fragments were collected for different studies as below. Animals were euthanized by exsanguination (full bleed) under deep anesthesia by cardiac puncture. The anesthetic protocols included ketamine (100mg /mL) combined with acepromazine (10 mg /mL) at a ratio of 9:1 (dose of 0.15 mL/100 g body weight) [14]. Blood samples were collected by cardiac puncture without anti-coagulant.

### Ethics statement

This study was carried out in full accordance with all international and Brazilian accepted ethic guidelines and was approved by the Oswaldo Cruz Foundation Ethics Committee on Animal Use [CEUA-*Comissão de Ética no Uso de Animais*, under protocols CEUA: LW81/12 for *N*. *Squamipes* and CEUA: 32/2012 for Swiss mice). CEUA follows the Brazilian national guidelines recommended by CONCEA (*Conselho Nacional de Controle em Experimentação Animal*).

Animals (*N*. *Squamipes*) were captured under authorization of Chico Mendes Institute for Biodiversity and Conservation of the Brazilian Government (ICMBIO, authorization number 13373). All procedures with *N*. *squamipes* were carried out in the field in accordance with biosafety standards level three. Biosafety techniques and personal safety equipment were used during all procedures according to the Brazilian Ministry of Health recommendations [15]

Mice experimentally infected and uninfected controls were monitored daily for survival and well-being status (home cage evaluation, body condition, skin lesions, mobility and other general conditions) [16]. No animals died prior to the experimental endpoints (55 days or 120 days for acute and chronic phases, respectively).

### Histopathology and histoquantitative analyses

Liver samples from uninfected and infected *N*. *squamipes* (3 animals/group) were removed from the right lobe of the organ and divided into approximately 5 mm^3^ fragments, which were immediately fixed in 4% paraformaldehyde in buffered phosphate, pH 7.3, 0.1 M overnight at 4°C [17]. Next day, the specimens were transferred to a 0.1M phosphate buffer solution, pH 7.3 and kept in this solution at 4°C for further histological processing. Samples were then dehydrated, embedded in glycolmethacrylate resin (GMA) (Leica Historesin Embedding Kit, Leica Biosystems, Heidelberg, Germany) as before [17] and cut at 3 μm thick sections using a Leica microtome RM2155. Three sections of each organ were obtained at an interval of 300 μm between sections to ensure analysis of different granulomas. Sections were stained with hematoxylin-eosin (Sigma-Aldrich, USA) or Gomori’s trichrome for qualitative and quantitative evaluation of granulomas and inflammatory processes.

Slides were scanned using a *3D Scan Pannoramic Histech* scanner (3D Histech Kft. Budapest, Hungary) connected to a computer (Fujitsu Technology Solutions GmbH, Munich, Germany). With the help of the *Pannoramic Viewer 1*.*15*.*2 SP2 RTM* software (3D Histech kft.), section areas (total of 111 mm^2^), granulomas and inflammatory infiltrates were demarcated and quantified.

### Quantitative analyses of parasite eggs

To evaluate the occurrence and distribution of parasite eggs in the liver of infected *N*.*squamipes*, the mean number of eggs/mm^2^ of tissue was investigated in HE-stained sections with a slide scanner (Pannoramic Histech 3D Scan). Quantitative studies were performed using the *Pannoramic Viewer* software (3D Histech kft.) as follows: the total number of eggs in each entire section (n = 3 sections/organ) from 3 animals was counted and divided by the sectional tissue area. A total of 725 eggs were counted in 111.79 mm^2^.

### LD staining

The formation of LDs was investigated in the hepatic tissue from both natural and experimental models by staining with Oil Red O (ORO) [1-(2,5-dimethyl-4-(2,5-dimethylphenyl) phenyldiazenyl azonapthalen-2-ol] (Sigma-Aldrich, USA) [18]. Liver fragments were fixed in a 4% paraformaldehyde solution overnight at 4°C, transferred to 0.1M phosphate buffer, pH 7.3 and stored at 4°C for further processing. Before microtomy, samples were kept in 30% sucrose solution in phosphate buffer, pH 7.3, overnight at 4°C, immersed in cryoprotector medium (Tissue Tek® Fisher Scientific, cat. no. 14-373-65, Massachusetts, USA), frozen and cut in a cryostat (Leica Biosystems, CM 1850 model, Heidelberg, Germany). Five-micrometer serial sections were obtained at intervals of 20 μm to avoid LD recounting. Sections on glass slides (Colorfrost® Fisher Scientific, cat. no. 14-373-65, Massachusetts, USA) were circled with a hydrophobic pen (Vector Laboratories, California, USA) and stained as the following steps: immersion in 100% propylene glycol (Vetec, Rio de Janeiro, Brazil) for 2 min; 5% ORO for 6 min; 85% propylene glycol for 1 min; two washes with distilled water and counterstaining with modified Harris hematoxylin (no alcohol) for 3 min to highlight the hepatocyte nuclei and mounted with 70% glycerol.

### Quantitative analyses of LDs by light microscopy

For quantitative evaluation of LDs in the liver, ORO-stained sections were analyzed with a slide scanner (Pannoramic Histech 3D Scan, 3D Histech kft., Budapest, Hungary). Quantitative studies were performed using the *Pannoramic Viewer* and *Histoquant* softwares (3D Histech kft.) as follows: 5 randomly areas of the liver tissue measuring 100,000 μm^2^ per area were outlined in each section (n = 3 sections/animal), performing a total analysis of ​​1,500,000 μm^2^ of tissue area per animal. Areas were randomly chosen in the liver tissue. In infected tissues, areas were not marked over inflammatory infiltrates or granulomas. In the demarcated areas, LD quantification within hepatocytes was performed by counting all LDs and all nuclei of hepatocytes, considering one nucleus/cell. The number of LDs per hepatocyte was obtained by dividing the total number of LDs per the total number of nuclei of hepatocytes.

### Transmission electron microscopy (TEM)

Liver fragments were fixed as before [19] in a mixture of freshly prepared aldehydes (1% paraformaldehyde and 1.25% glutaraldehyde) in 0.1 M sodium cacodylate buffer for 4h at RT. Samples were post-fixed in 1% osmium tetroxide in Sym-Collidine buffer (pH 7.4) for 2h at RT. After washing with sodium maleate buffer (pH 5.2), samples were stained en bloc in 2% uranyl acetate in 0.05 M sodium maleate buffer (pH 6.0) for 2h at RT and washed in the same buffer as above before dehydration in graded ethanols and infiltration and embedding with a propylene oxide-Epon sequence (Eponate 12 Resin; Ted Pella, Redding, CA, USA). After polymerization at 60°C for 16h, thin sections were cut using a diamond knife on an ultra-microtome (Leica, Baden-Württemberg, Germany). Sections were mounted on uncoated 200-mesh copper grids (Ted Pella) before staining with lead citrate and viewed with a transmission electron microscope (CM 10; Philips, or Tecnai–G2-20-FEI 2006, Eindhoven, the Netherlands) at 60 kV.

### TEM quantitative analyses

Quantitative analyses were performed on randomly taken electron micrographs of liver tissue from *N*. *squamipes*. The following parameters were measured: i) number of LDs per sectional area of liver tissue; ii) diameter of LDs. A total of 34,000 μm^2^ (17,000 from uninfected and 17,000 from infected animals) scanned from 54 electron micrographs was analyzed. For evaluation of LD diameters, a total of 650 LDs were counted and measured. All analyses were performed using the *ImageJ*® software (National Institutes of Health, Bethesda, MD, USA).

### Raman spectroscopy

Liver fragments, fixed as for histopathology, were analyzed by Raman spectroscopy. The Raman spectra were obtained using a spectrometer RFS 100 FT- Raman Bruker (Bruker Optik GmbH, Ettlingen, Germany) equipped with a Ramscope Raman microscope and Ge detector, cooled down with liquid nitrogen. The excitation line utilized was 1064 nm, using Nd: YAG laser. Measurements were made with a resolution of 4 cm^-1^, laser power of 75 mW, acquisition time of approximately 15 min, corresponding to 1024 accumulations; the spectral range used was from 3500 to 400 cm^-1^. The spectra were collected in triplicate (from 3 fragments per animal, n = 3 animals per group) and the verification of non-destruction of the sample in the laser impact region was made. In this case, there were performed instrumental replicates, which corresponded to obtaining in triplicate spectra of the same sample. The spectra were acquired using Opus 6.0 software (Bruker Optik GmbH, Ettlingen, Germany).

In order to get an overview of the spectra obtained in different hepatic tissues, exploratory analysis were performed using the Principal Components Analysis (PCA) and the Hierarchical Components analysis (HCA) by KNN (k-nearest neighbor). The PCA and HCA were performed in Matlab 7.10.0 (R2010a). Before the decomposition of the spectral matrix, data has been preprocessed to remove systematic variations. In this case, the spectra were corrected at the baseline and were normalized to unit length. The number of principal components was chosen based on the % of the total variance explained by the new components.

### MALDI-TOF mass spectroscopy

Matrix-assisted laser desorption/ionization time-of-flight mass spectrometry (MALDI-TOF-MS) experiments were performed using a pulsed nitrogen laser at 337 nm of a Shimadzu Biotech Axima Performance MALDI-TOF-MS at the Physics Department (UFJF, Brazil). The time-of-flight mass spectrometer consists basically of an electrostatic ion extraction system, a collimating electrostatic lens, a drift tube, an electrostatic mirror and a pair of microchannel plate (MCP) detectors, disposed in the chevron configuration (refletron detector) [20]. After extraction, ionic molecular fragments travel through the flight tube until reaching the MCP.

For a correct instrument calibration, it is essential to deposit the calibrant very close to the sample spots, thus allowing obtaining a better mass accuracy. The calibrant used was alpha-cyano-4-hydroxycinnamic acid (CHCA), dissolved in (50:50 v:v) water milli-Q quality/acetonitrile with 0.1% TFA (trifluoroacetic acid) in a concentration of about 5×10^−2^ mol/L. This enables the operator to internally calibrate the sample spectrum using the calibrant peaks. The matrix was also CHCA. For MALDI-TOF-MS measurements, 50 μm cryostat sections from infected and uninfected livers were placed in the stainless steel multiprobe, covered with the matrix solution and inserted into the mass spectrometer. The instrument was set in high-resolution in the positive reflector ion mode and the spectra were taken from 100 to 600 *m/z*. A typical laser power is 80. The experimental setup included an automatic sample manipulator, where 200 scans were accumulated with 20 repetitions each. Mass spectra of lipid species were identified according to lipid MAPS structure database (LIPID MAPS® at *www.lipidmaps.org*).

### Biochemical determinations

To evaluate the serum lipid profile and enzymes reflecting liver function, blood samples were collected from *N*. *squamipes* by cardiac puncture without anti-coagulant, centrifuged at 3000 rpm for 10 min to obtain sera and analyzed in a Roche Cobas Mira Plus Chemistry Analyzer (Roche Diagnostics®, IN, USA). For biochemical evaluations, assay Kits (Bioclin, Quibasa, Brazil) were used according to the manufacturer's instructions. Animals were not subjected to prior fasting. A total of 26 samples were evaluated from *N*. *squamipes* (13 from uninfected and 13 from infected animals). These samples included 6 samples from the same animals used for histological and ultrastructural analyses and other 20 samples obtained from previous expeditions.

### Statistical analysis

Two Way ANOVA followed by Tukey's post-test was used for quantitative analysis of LDs in histological sections from different groups of infected and non-infected animals (natural and experimental infection). The *Student t test* was used for quantitative ultrastructural analysis of LDs and biochemical analysis in *N*. *squamipes*, in which 2 groups (uninfected and infected) were compared. All analyses were performed using *Prism* 1.6 (Graphpad Software, San Diego, CA) software. The significance level was set at *p <0*.*05*. Multivariate data analysis was used for evaluation of the Raman spectra.

## Results

### Natural *S*. *mansoni* infection induces a typical granulomatous inflammatory response in the liver

First, the parasitism of *S*. *mansoni* in naturally infected *N*. *squamipes* was evaluated. Counting of recovered worms after perfusion of the portal-hepatic system showed 10.7 ± 1.7 recovered worms/infected host (mean ± SEM, n = 3 animals), which demonstrate the susceptibility of these animals to the infection [8].

**Fig 1:**
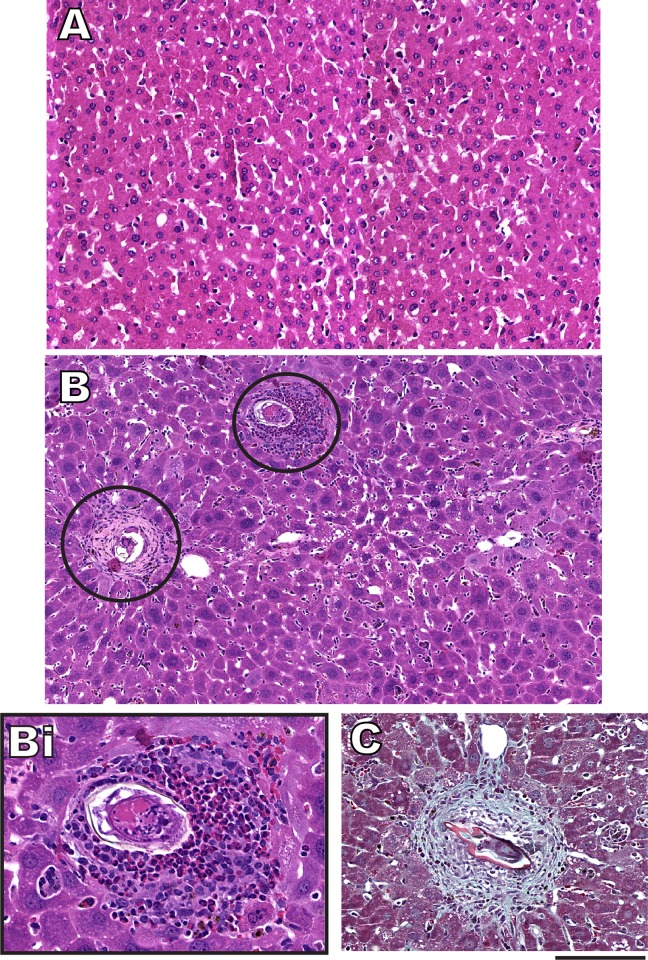
**Representative histological images of the liver from uninfected (A) and naturally *S*. *mansoni*-infected *N*. *squamipes* (B).** A normal hepatic tissue is seen in (A) while typical granulomas at different developmental stages (circles) are observed in (B). One mature granuloma characterized by a central parasite egg surrounded by a dense population of inflammatory cells is shown in high magnification in (Bi). Eosinophils with characteristic acidophilic cytoplasm and mononuclear cells are clearly observed. In (C), the layer formed by collagen fibers is seen at the outer zone of the granuloma. Liver fragments (n = 3 animals from each group) were fixed in buffered paraformaldehyde, embedded in glycolmethacrylate resin and cut into 3 μm-thick sections, which were stained with hematoxylin-eosin (A, B) or Gomori’s trichrome (C). Scale bar, 60 μm (A, B); 120 μm (Bi); 100 μm (C).

During the infection with *S*. *mansoni*, eggs become trapped in the liver and elicit a granulomatous inflammation characterized by accumulation of immune cells such as eosinophils, lymphocytes and macrophages intermixed with collagen fibers around the eggs [3]. Therefore, we sought to identify granulomas in the hepatic tissue of naturally infected *N*. *squamipes*. To study liver histopathology, we used a histological approach that combines optimal fixation and processing with a plastic resin (glycolmethacrylate) embedding [17]. This approach allows increased tissue resolution and optimal visualization and quantification of inflammatory processes [17]. Moreover, we studied the distribution of granulomas in entire histological sections by using a whole slide scanner which enables visualization of large areas of tissue. Our histopathological analyses showed that the granulomatous inflammatory response around the parasite eggs of *S*. *mansoni*-infected *N*. *squamipes* is a well-characterized lesion as previously documented for this rodent [21, 22]. Granulomas in different developmental phases including involutional types were clearly observed in the liver and inflammatory cells composed of eosinophils and mononuclear cells were seen around deposited eggs (Fig 1). Quantitative analyses demonstrated that the percentage of hepatic tissue taken by inflammatory granulomatous processes represented just 5.04 ± 0.46% (mean ± SEM, n = 3 animals) of the organ in infected *N*. *squamipes* while the number of parasite eggs in the liver was 1.74 ± 0.43/mm^2^ of tissue.

### Natural *S*. *mansoni* infection elicits excessive hepatic LD formation and increases serum lipid profile

**Fig 2:**
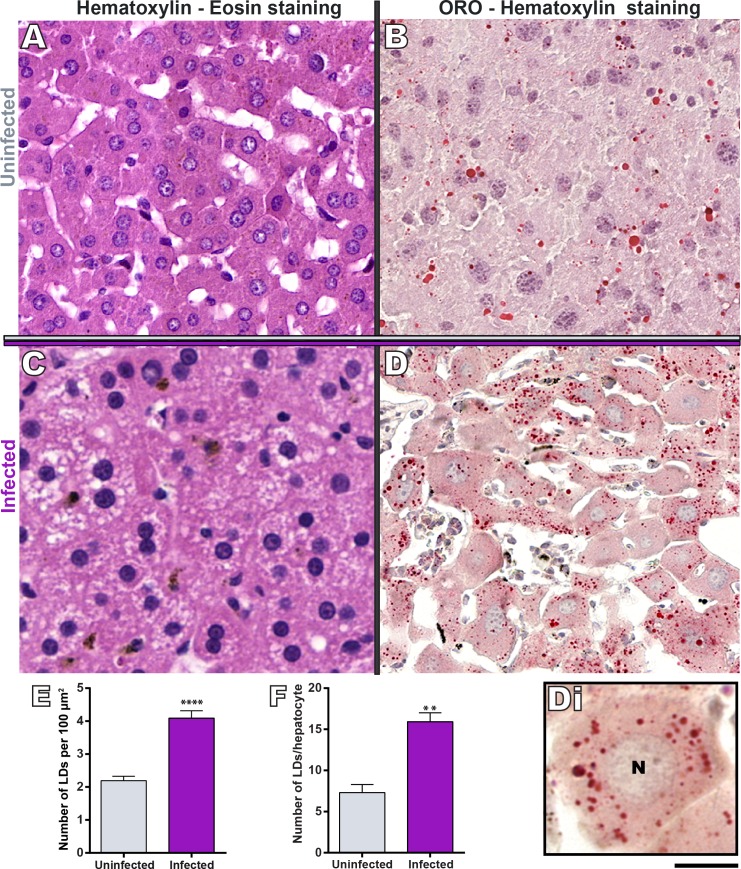
Excessive LD accumulation occurs in the liver of naturally *S*. *mansoni-*infected *N*. *squamipes*. In (A and C), the hepatic tissue shows putative LDs, negatively stained with alcoholic hematoxylin-eosin. (B and D), ORO staining confirms the presence of numerous LDs seen as round organelles (stained in red) and distributed within hepatocytes. In (Di), a hepatocyte is seen in high magnification. (E, F) Quantitative analyses of LD numbers in the hepatic tissue. ORO staining was performed on cryosections from liver fragments fixed in buffered paraformaldehyde. LD quantifications were done in a slide scanner using *Pannoramic Viewer* and *Histoquant* softwares. A total of 1,500,000 μm^2^ of tissue area was evaluated per animal, with a total of 4,500,000 μm^2^ of tissue area analyzed per group (n = 3 animals). Data represent mean ± S.E.M. ****P < 0.0001, ** *P* < 0.004 versus uninfected group. Scale bar, 40 μm (A,D); 50 μm (B); 75 μm (C); 8 μm (Di).

By studying the liver histopathology of *N*. *squamipes* naturally infected with *S*. *mansoni*, we noticed the occurrence of a high number of round, negatively stained structures resembling LDs (compare Fig 2A with 2C). Because LDs are solubilized by commonly used alcohol-based stainings [18, 23] such as hematoxylin-eosin applied to our histopathogical analysis (Fig 2A and 2C), we next investigated the presence of LDs by using appropriate fixation with paraformaldehyde followed by staining of liver cryosections with a lipid probe–ORO. This stain enables clear visualization of LDs by both bright-field and fluorescence microscopy [18, 24] and it is extensively used for histologic evaluation of hepatic steatosis [25–27]. ORO staining confirmed the occurrence of numerous LDs in the hepatic tissue. These organelles were localized within hepatocytes and showed different sizes (Fig 2B and 2D). Next, we performed a comprehensive quantitative analysis to investigate the number of LDs per area of hepatic tissue using a slide scanner and *Pannoramic Viewer* and *Histoquant* softwares. A total of 1,500,000 μm^2^ of tissue area was evaluated per animal, with a total of 4,500,000 μm^2^ of tissue area analyzed per group (n = 3 animals). This analysis showed that LDs are consistently present in the liver of both uninfected and infected animals and that the liver of *S*. *mansoni*-infected *N*. *squamipes* showed increased LD numbers per tissue area compared to the control group (2.19 ± 0.13 and 4.08 ± 0.22 for uninfected and infected group respectively, mean ± SEM, *P*<0.0001) (Fig 2E). Next, the distribution of LDs within hepatocytes was investigated (Fig 2F). This analysis also demonstrated a significant increase of LD numbers per hepatocyte in infected compared to uninfected animals (7.31 ± 0.98 in the control versus 15.9 ± 1.08 in infected group, mean ± SEM, *P*< 0.004). Interestingly, parallel studies using an experimental model of *S*. *mansoni* in mice demonstrated no LD formation in the liver during both acute and chronic infection (S1 Fig).

To get more insights into the structural features of LDs accumulated in the liver of infected *N*. *squamipes*, we next used TEM. While LD imaging under light microscopy requires the use of specific lipid probes, ultrastructural observation does not require any additional labeling because LDs lack a true delimiting unit membrane structure, which enables unambiguous identification by TEM [28]. Thus, this unique LD ultrastructural feature distinguishes this organelle from all other cytoplasmic membranous organelles and vesicles that have an aqueous content surrounded by a phospholipid bilayer membrane [28, 29].

**Fig 3:**
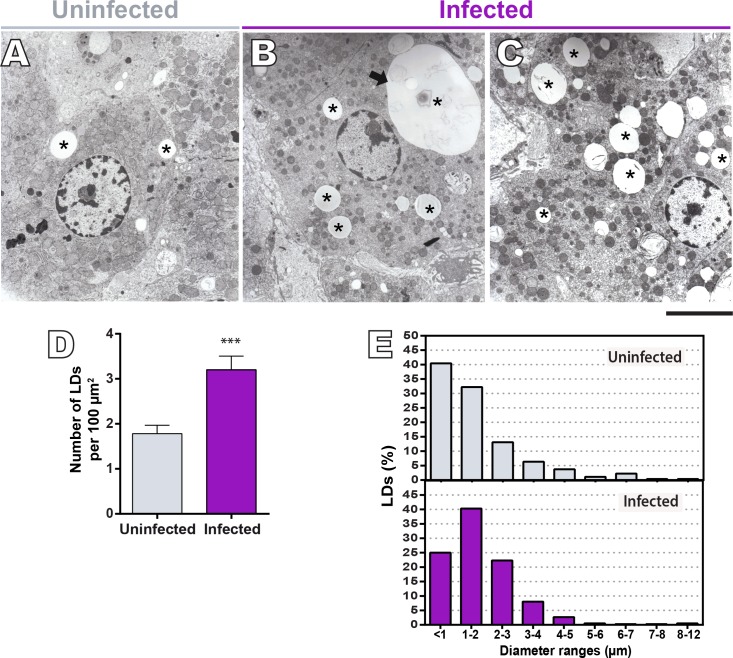
Ultrastructure of LDs formed in the liver of uninfected and naturally *S*. *mansoni*-infected *N*. *squamipes*. (A-C) Electron micrographs of the hepatic tissue reveal electron lucent LDs with varied sizes within hepatocytes in both control (A) and infected (B, C) livers. Note in (B), a giant LD (arrow) in the hepatocyte cytoplasm. (D) TEM quantitative analyses show a high number of LDs per tissue area (*P* < 0.002). The range of LD diameter is shown in (E). Scale bar, 9 μm (A-C). Liver fragments were fixed in a mixture of paraformaldehyde/glutaraldehyde and processed for TEM. Quantitative analyses were performed in a total of 34,000 μm^2^ of hepatic tissue (17,000 μm^2^ from uninfected and 17,000 μm^2^ from infected animals) using the software *ImageJ*. Data represent mean ± S.E.M. ***P < 0.0001 for infected versus uninfected group.

In both non-infected and infected groups, hepatocytes exhibit LDs as distinct, non-membrane bound cytoplasmic sites mostly seen as electron-lucent organelles (Fig 3A–3C). Quantitative EM analyses using the software *Image J* confirmed a significant increase of LD numbers per tissue area in infected liver compared to the uninfected group (1.78 ± 0.19 in the control versus 3.20 ± 0.30 in infected group, mean ± SEM, *P*< 0.0002) (Fig 3D). Moreover, TEM clearly revealed that LDs greatly varied in size, with diameters ranging from less than 1 to 12 μm (Fig 3A–3C and 3E).

**Fig 4:**
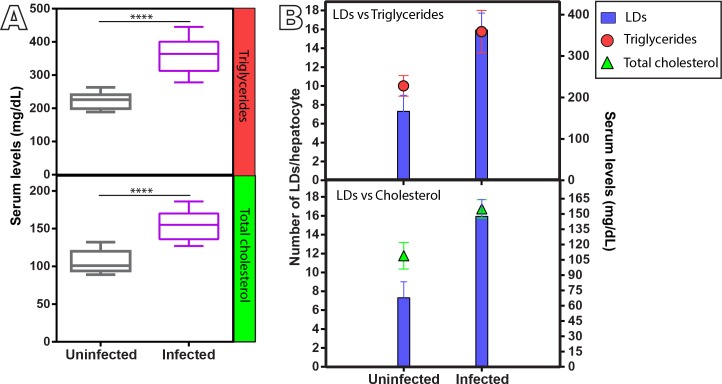
Serum lipid profile of uninfected and naturally *S*. *mansoni*-infected *N*. *squamipes*. (A) The levels of total cholesterol and triglycerides are significantly increased in the infected group in comparison to the uninfected animals (****P < 0.0001, n = 13 animals per group). (B) Serum lipids increase proportionally to the increase of LD numbers in the liver. LDs were counted in the liver after ORO staining as shown in Fig 2. Data represent minimum-median-maximum (A) and mean ± S.D (B).

Next we evaluated if the serum lipid profile was also altered during the natural *S*. *mansoni* infection. Biochemical analyses from a total of 26 animals (13 from uninfected and 13 from infected) showed that the levels of both total cholesterol and triglycerides were significantly increased in infected animals compared to the uninfected group (Fig 4A). This increase was proportional to the increase of LD numbers in the liver (Fig 4B).

Altogether, our results showed, for the first time, that the wild reservoir *N*. *squamipes* accumulates lipids in response to the natural infection with *S*. *mansoni*.

### Raman spectroscopy reveals high content of unsaturated lipids in the liver of *N*. *squamipes*

**Fig 5:**
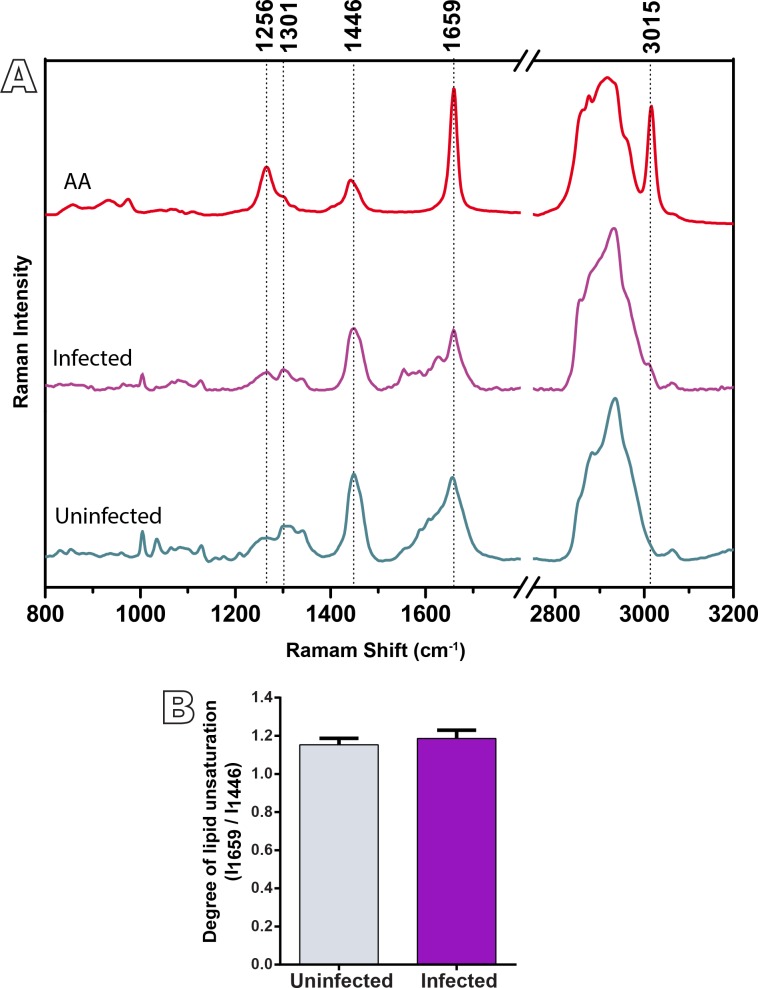
Raman spectra reveal high content of polyunsaturated fatty acids (PUFAs) in both uninfected and infected liver of *N*. *squamipes*. (A) The band at 3015 cm^-1^ is characteristic of PUFAs and is more prominent in arachidonic acid (AA) when compared to other PUFAs. The degree of unsaturation is shown in (B). Liver fragments of *N*. *squamipes* naturally infected and uninfected were fixed and analyzed by Raman spectroscopy without labeling. AA spectrum was obtained from pure AA (catalog number A3555, Sigma-Aldrich) diluted in ethanol. Data are representative of 3 independent experiments.

To explore the molecular properties of lipids within the liver of *N*. *squamipes*, we next used Raman spectroscopy. This technique provides information about the chemical composition of the biological sample, with the advantages of minimal sample preparation, without need of labeling and free from water interference due to the low cross-section of water for the Raman effect [30]. The result is showed by the Raman spectrum, where frequencies of characteristic Raman bands provide information of the composition of samples and the intensity of Raman bands is proportional to the relative concentration of a compound [30]. Fig 5A shows the mean Raman spectra (average of triplicates) found in the animals studied. The Raman spectra of hepatic tissues showed vibrational modes of lipids, compounds of interest in this study, but it has also been observed vibrational modes of other compounds, such as proteins and heme group. The saturated lipid characteristic bands are observed at 1301 and 1446 cm^-1^ and they are assigned to CH_2_ deformation vibrations. The bands in the region of 1659 cm^-1^, 1256 and 3015 cm^-1^ reveal the presence of unsaturation. Other Raman bands in the tissue spectra may be assigned to the presence of proteins and heme group [31].

The visual analysis of average Raman spectra yielded information in relation to the lipid composition of the hepatic tissues. The main difference between the Raman spectra of samples of uninfected and infected tissues in relation to vibrational modes of lipids was the band observed at 3015 cm^-1^ (Fig 5A). This band is attributed to the CH stretching vibration mode of the chemical group -C = C-H, characteristic of polyunsaturated fatty acids (PUFAs) and is more prominent in omega-6 arachidonic acid (AA) (20:4) when compared to other PUFAs [31] (Fig 5A). The shoulder at 3015 cm^-1^ appeared only in the Raman spectra of infected *N*. *squamipes*, while for the uninfected group, this band was not observed; this result shows that the AA concentration is larger for the infected group, and indicates that any low concentration of AA present in the uninfected group could not be detected by the Raman technique.

The ratio of the Raman bands at 1659 and 1446 cm^-1^ have provided information on the lipid composition in the liver and may be used to calculate the degree of unsaturation. Thus, from each spectrum obtained, we next calculated the intensity ratio of the two bands. A higher intensity ratio indicating high unsaturation was found for both uninfected and infected animals (Fig 5B), but no statistically significant difference was found when the 2 groups were compared.

### A high store of AA is found in the infected liver

To get more insights into the lipid composition of the liver from *N*. *squamipes* and confirm the presence of AA, we next used MALDI-TOF-MS, which allows direct and precise identification of a wide range of endogenous molecular species such as lipids [32], proteins [33] and peptides [34], without any labeling.

**Fig 6:**
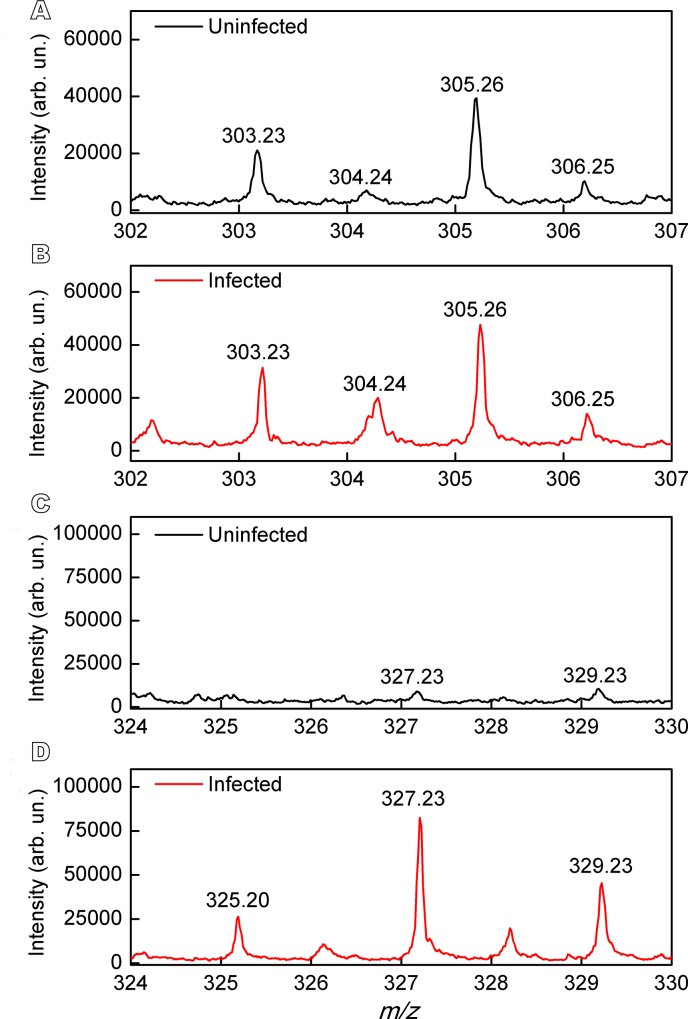
MALDI-TOF mass spectra of liver tissues reveal high concentration of arachidonic acid (AA) in naturally *S*. *mansoni*-infected *N*. *squamipes*. (A, B) Mass spectra, from 301 to 307 m/z range showing peaks attributed to sodium adduct of the linoleic acid [M + Na]^+^ (*m/z* 303.03), AA (*m/z* 304.24) and sodium adduct of the oleic acid [M + Na]^+^ (*m/z* 305.26). (C, D) Mass spectra from 324 to 330 *m/z* range. The peak observed at 327 *m/z* represents sodium adduct of the AA [M + Na]^+^. Liver sections from uninfected and naturally *S*. *mansoni*-infected *N*. *squamipes* (n = 3 sections from each group) were cut on a cryostat (50 μm thickness) and analyzed without any labeling.

In Fig 6A and 6B, the liver tissue mass spectra of lipid species are presented for *N*. *squamipes* samples for a mass range from *m/z* 302 to 307, which correspond mostly to PUFAs. In both uninfected and infected liver samples, the following PUFAs were identified within this range: AA (20:4) and linoleic acid (LA) (18:2), both omega-6. Moreover, oleic acid (OA) (18:1), which is a monounsaturated fatty acid, was also detected. The peak *m/z* 304.24 attributed to the ion [M]^+^ (C_20_H_32_O_2_) refers to AA. The other peaks are attributed to the presence of protonated molecules [M + H]^+^ and/or sodium adduct [M + Na]^+^, the most often chemical events observed during MALDI-TOF-MS analyses in the positive ion mode [35–37]. Thus, the peak *m/z* 303.23 can be attributed to the ion C_18_H_32_NaO_2_ [M + Na]^+^ and refers to the sodium adduct of the omega-6 LA while the peak *m/z* 305.25 refers to the [M + Na]^+^ of the OA (C_18_H_34_NaO_2_) [37]. We also observed sodium adduct to the AA [M + Na]^+^ (C_20_H_32_NaO_2_), revealed by a of peak *m/z* 327.23 (Fig 6C and 6D).

When we compared the area values of AA peaks between uninfected and infected animals (n = 3 sections for each group), we found that the peak area of *m/z* 304.24, referring to AA is twice higher in infected (Fig 6B) compared to the uninfected group (Fig 6A) (uninfected = 1312 and infected = 3104). The peak area of *m/z* 327.23 in the infected group (Fig 6D) is more than 10 times greater compared to uninfected *N*. *squamipes* (Fig 6C) (uninfected = 3135 and infected = 45145). We also detected increased area for *m/z* 303.23 (LA) and 305.25 (OA) peaks (respectively, 1.7 and 1.8 times higher in infected compared to uninfected animals) (Fig 6A and 6B).

Altogether, our findings using both Raman and MALDI-TOF-MS identified high stores of PUFAs, markedly AA, accumulated in the liver from naturally S. *mansoni*-infected *N*. *squamipes*.

### Parameters reflecting liver function do not change in naturally infected animals

As noted, *Schistosomiasis mansoni* is a parasitic liver disease, which causes hepatic metabolic disturbances [1]. It is well documented that the levels of enzymes reflecting liver function such as transaminases (ALT/AST) are increased in response to both experimental [38–40] and human [41] infection. Thus, next, we evaluated the levels of these enzymes in the serum of *N*. *squamipes*. In spite of a well-characterized granulomatous response found in the liver of these animals (Fig 1), enzyme levels were not altered in infected compared to uninfected animals (Table 1).

**Table 1:** Serum transaminases of uninfected and *S*. *mansoni*-infected *N*. *squamipes*.

Parameters	Animals		*P* value**
	Uninfected (n = 13)	Naturally infected (n = 13)	
AST (U/L)	87 ± 11	91 ± 11	0.4
ALT (U/L)	65 ± 6	65 ± 6	> 0.9
De Ritis ratio*	1.4 ± 0.1	1.4 ± 0.1	> 0.9

Serum samples were taken from *N*. *squamipes* (at day one of capture after infection confirmation) and their respective uninfected controls from the same age.

AST indicates Aspartate Aminotransferase; ALT, Alanine Aminotransferase.

*De Ritis ratio was calculated as [AST/ALT] and indicates the degree of hepatocelular damage. Data are presented as means ± S.D.

** test *t* student between means of each group in each row.

Taken together, we identified that the natural *S*. *mansoni* infection in *N*. *squamipes* induces liver steatosis in the absence of liver functional impairment, a finding with potential implication to the adaptation of this wild reservoir to the infection.

## Discussion and Conclusions

Intracellular excessive accumulation of lipids and subsequent formation of LDs in the cytoplasm of hepatocytes characterizes a condition termed hepatic steatosis [42]. Here we identified, for the first time, that a hepatic steatosis and a significant increase of serum lipids occur in response to the natural *S*. *mansoni* infection of the wild reservoir *N*. *squamipes*. Intriguingly, lipid accumulation was identified in parallel to low incidence of granulomatous inflammatory processes and in absence of liver functional harm.

Previous studies have demonstrated that *N*. *squamipes* is highly susceptible to the experimental *S*. *mansoni* infection [11, 43–45] and that both experimental and natural infection of this wild reservoir reproduce the granulomatous reaction observed in experimental infections in other models, such as mice [21, 22]. Moreover, *S*. *mansoni* isolated from *N*. *squamipes* is susceptible to the therapeutic effects of praziquantel [46], the most used chemotherapy to treat human schistosomiasis [1].

Our present data demonstrate that the natural *S*. *mansoni* infection of *N*. *squamipes* interferes more with the quantity than with the quality of the pathological inflammatory responses in the liver. This aspect is the main difference reported so far in the literature between the natural infection in *N*. *squamipes* and the experimental *S*. *mansoni* infection in mice [21]. In fact, we expanded this observation by demonstrating here that the granulomatous response reaches just 5% of the liver in the natural infection of *N*. *squamipes* while this organ is greatly affected by both the murine experimental and human *S*. *mansoni* infections (reviewed in [4, 47, 48]). Interestingly, the low intensity of the granulomatous response found in the natural infection occurred even in the presence of high number of parasite eggs lodged in the liver, which was comparable to the experimental infection in mice (data not shown).

We identified here that a low incidence of granulomas in the liver of the naturally infected *N*. *squamipes* coexists with a high storage of lipids in the form of LDs. Several classes of lipids, including neutral lipids, cholesterol, and phospholipids, make up LDs [49]. Increase in LD numbers was paralleled by proportional increase of serum lipids (total cholesterol and triglycerides) (Fig 4), thus demonstrating that the natural schistosomiasis mansoni cohabits with an elevated lipid store. This is a remarkable difference that we found when the natural infection in *N*. *squamipes* was compared to the experimental infection in mice in which there was no LD formation in the liver during both acute and chronic phases of the infection (S1 Fig).

Is the lipid accumulation, detected during the natural infection with *S*. *mansoni*, hepatoprotective? Our data showing a correlation between high lipid content and low prevalence of hepatic granulomas suggest that lipid accumulation/metabolism might be protecting this organ against the natural *S*. *mansoni* infection. Indeed, by assessing the levels of ALT/AST, which reflect liver function, we found no alterations in the natural *S*. *mansoni* infection in contrast to both experimental [38–40] and human [41] schistosomiasis mansoni.

Histological analyses (Fig 2) and Raman spectroscopy (Fig 5) revealed a considerable number of LDs and high content of PUFAs in the liver from both uninfected and naturally infected *N*. *squamipes*. This means that before acquiring the infection, the rodent *N*. *squamipes* has already a substantial level of hepatic lipids, which additionally increase significantly after infection resulting in hepatic steatosis. Thus, we can speculate that by storing PUFAs from its diet, this omnivorous wild reservoir would be “prepared” for life-long infections with *S*. *mansoni* without any consequences on its lifespan. As noted, *N*. *squamipes* presents a well-balanced relationship with the parasite and life-long *S*. *mansoni* infections (and re-infections) seem not to affect its lifespan [8]. Potential beneficial effects of lipids include modulation of the immune system (reviewed in [50, 51]), and increased liver regeneration as observed during the experimental *S*. *mansoni* infection with concurrent high-fat diet in Swiss mice [52].

One interesting finding of the present work was the presence of higher levels of omega-6 AA in the liver from naturally *S*. *mansoni*-infected animals compared to uninfected controls, as markedly revealed by both Raman and MALDI-TOF-MS (Figs 5 and 6, respectively). Tissue AA pools originate from the diet and from desaturation-elongation of dietary LA, a process that predominantly occurs in the liver [53]. Therefore, this organ has a central role in the genesis and metabolism of AA [53]. Interestingly, our present findings using MALDI-TOF-MS demonstrated not only consistent pools of AA but also of LA in the liver of *N*. *squamipes*, with increased concentration of both molecules in infected animals (Fig 6).

AA is an essential fatty acid present in the phospholipids of cell membranes [54] and it is also stored within LDs mainly in cells from the immune system (reviewed in [23, 55]). It is a precursor of a large family of pro-inflammatory eicosanoids, including the prostaglandins and leukotrienes through enzymes such as cyclooxygenase and lipoxygenase [54].

It is well documented that accumulation of LDs in host cells, mainly in macrophages infected with intracellular parasites, may favor parasite survival (reviewed in [56]). It is proposed that newly formed, parasite-induced LDs may serve as lipid sources for parasite growth and also produce inflammatory mediators (through the metabolism of AA), which potentially act in the host immune response deactivation [56]. Therefore, it seems, at the first view, contradictory that naturally *S*. *mansoni*-infected *N*. *squamipes* with a high content of both LDs and AA develop low incidence of granulomatous inflammatory responses. However, the scenario established for *S*. *mansoni* infection is quite different compared to infections with intracellular parasites that have an obligate intracellular existence within the parasitophorous vacuole in which they multiply thus causing the infection (reviewed in [57]).

The experimental *S*. *mansoni* in mice did not trigger LD formation in hepatocytes (S1 Fig) nor did induce AA accumulation in the liver (data not shown) in response to the acute or chronic infection. During the natural Schistosomiasis mansoni, hepatic steatosis and high AA stores seem much more related to host protection. This might be explained by the fact that AA is also an schistosomicide with significant therapeutic and safe effects in both experimental [58] and human schistosomiasis mansoni [59]. The killing effect of AA on *S*. *mansoni* parasites is due to its ability to activate the parasite tegument-bound neutral sphingomyelinase, with subsequent hydrolysis of the apical lipid bilayer sphingomyelin molecules, thus allowing hostile access of the immune system, including antibodies [60–62]. Therefore, the identification of a high pool of hepatic AA during the natural *S*. *mansoni* may have implications in the parasite burden, although we cannot rule out the possibility that AA could be metabolized into pro-inflammatory molecules.

In conclusion, this work demonstrates that the natural infection of the wild reservoir *N*. *squamipes* with *S*. *mansoni* leads to hepatic steatosis in the absence of liver functional harm and that accumulation of lipids, markedly PUFAs, in the liver coexists with low incidence of granulomatous inflammatory responses, implying a protective role for PUFAs, particularly AA, in the pathogenesis of the disease, and thus supporting the natural adaptation of *N*. *squamipes* physiology to the *S*. *mansoni* infection.

## Supporting Information

S1 Fig Quantitative analyses of LD numbers in the hepatic tissue from naturally and experimentally infected animals. Liver fragments were fixed in buffered paraformaldehyde and stained with ORO. LD quantifications were performed in a slide scanner using *Pannoramic Viewer* and *Histoquant* softwares. A total of 1,500,000 μm^2^ of tissue area was evaluated per animal, with a total of 4,500,000 μm^2^ of tissue area analyzed per group (n = 3 animals). ****P < 0.0001. Data represent mean ± SEM.
(TIF)

## Abbreviations

LD: lipid droplet
PUFAs: polyunsaturated fatty acids
TEM: transmission electron microscopy
MALDI-TOF-MS: matrix-assisted laser desorption/ionization time-of-flight mass spectrometry
AA: arachidonic acid
LA: linoleic acid
OA: oleic acid
ALT: alanine transaminase
AST: aspartate transaminase
