# Supplementary figures and images for: Natural Schistosoma mansoni Infection in the Wild Reservoir Nectomys squamipes Leads to Excessive Lipid Droplet Accumulation in Hepatocytes in the Absence of Liver Functional Impairment
Source: PLoS One. 2016 Nov 23;11(11):e0166979. doi: 10.1371/journal.pone.0166979 (PMC5120838; doi:10.1371/journal.pone.0166979)

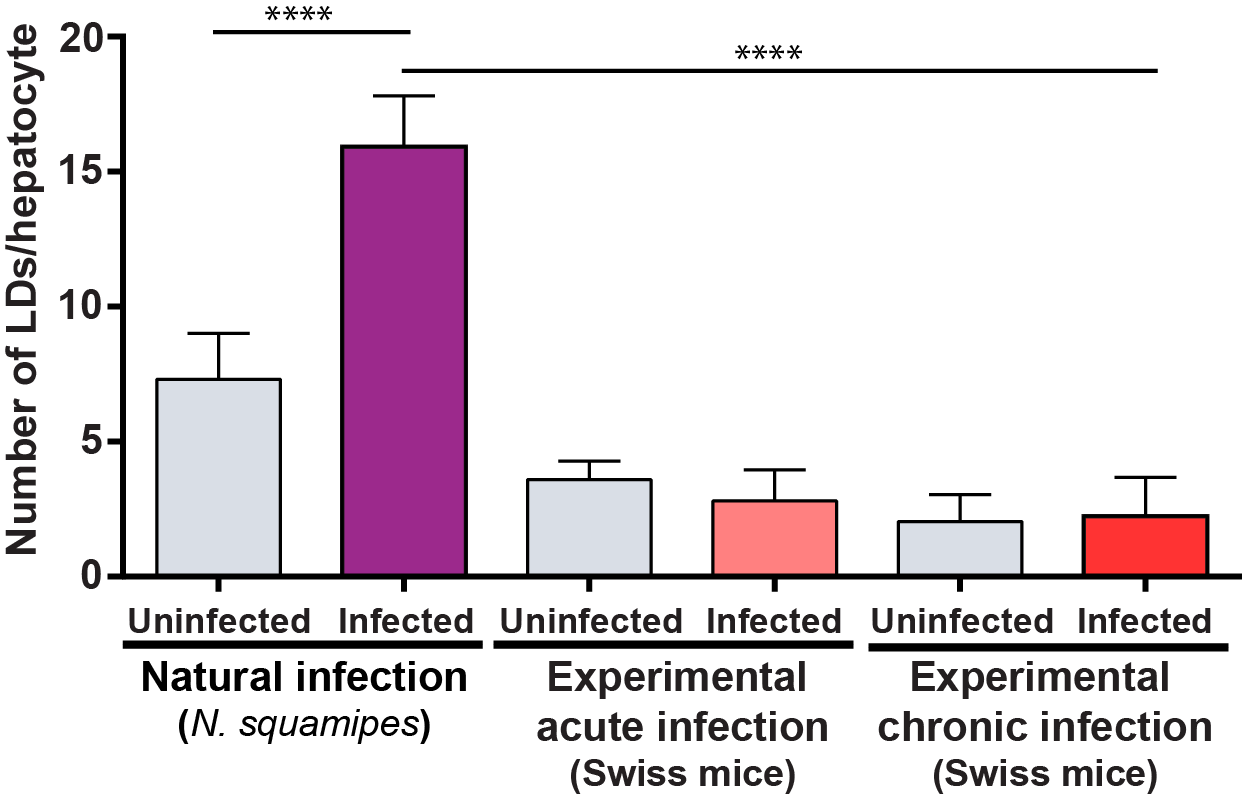

Supplement: S1 Fig — Liver fragments were fixed in buffered paraformaldehyde and stained with ORO. LD quantifications were performed in a slide scanner using Pannoramic Viewer and Histoquant softwares. A total of 1,500,000 μm2 of tissue area was evaluated per animal, with a total of 4,500,000 μm2 of tissue area analyzed per group (n = 3 animals). ****P < 0.0001. Data represent mean ± SEM. (TIF) [file pone.0166979.s001.tif]
